# Temperature-Enhanced Ordering in Plate-like Semicrystalline Block Copolymer Single-Crystal Suspensions Studied by Real-Time SAXS/WAXS

**DOI:** 10.1021/acs.langmuir.4c03853

**Published:** 2025-02-21

**Authors:** Enyi Chi, Haiying Huang, Fajun Zhang, Tianbai He

**Affiliations:** † State Key Laboratory of Polymer Physics and Chemistry, Changchun Institute of Applied Chemistry, Chinese Academy of Sciences, Changchun 130022, P. R. China; ‡ University of Chinese Academy of Sciences, Beijing 100049, P. R. China; § Institut für Angewandte Physik, Universität Tübingen, Auf der Morgenstelle 10, 72076 Tübingen, Germany

## Abstract

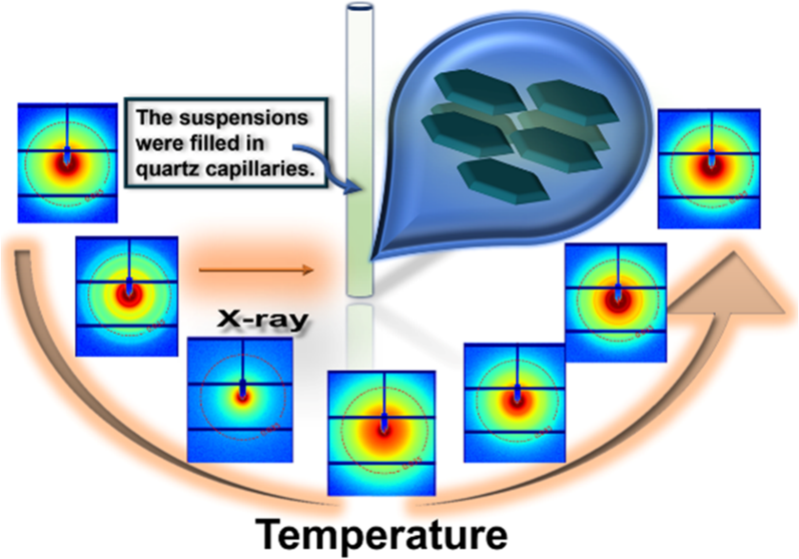

Self-assembly of hard platelet colloids into liquid crystalline phases is typically driven by entropy, making them less sensitive to temperature changes. However, soft interaction potentials often exist in real colloidal systems, which can lead to temperature-sensitive phase transitions. Despite significant progress in understanding phase behavior in the past 2 decades, studies on temperature-dependent phase behavior remain rare, and there is limited knowledge about how soft interactions influence phase transitions upon temperature changes. In this work, we investigated a platelet colloid system in isotropic and nematic phases using small- and wide-angle X-ray scattering techniques (SAXS/WAXS) and polarized optical microscopy (POM). The system consisted of polystyrene-*block*-poly(l-lactide) (PS-*b*-PLLA) block copolymer single crystals (BCSCs) with varying sizes dispersed in *p*-xylene. These crystals were truncated lozenge-shaped with effective diameters of 500 and 1000 nm and a uniform dry thickness of 18.0 nm. The ordering behaviors of BCSC500 in the isotropic phase and BCSC1000 in the N phase were monitored through SAXS/WAXS during heating, quenching, self-seeding, crystal growth, and final quenching. Enhanced ordering, specifically face-to-face correlation, was observed during heating prior to crystal melting. For BCSC500, ordering emerged at 105 °C during heating. In the case of BCSC1000 in the N phase, ordering was enhanced with increased heating and reached up to the ninth order of correlation peaks, indicating the formation of lamellar domains within the N phase. After seeding and crystal growth, both systems exhibited ordering. However, during the final cooling to room temperature, ordering disappeared for BCSC500 but persisted for BCSC1000. POM observations revealed that for both systems, initial heating resulted in a decrease in overall brightness; however, enhanced nematic domains or tactoids emerged prior to melting. Subsequent thermal treatments did not induce noticeable changes in the observed order. While both techniques revealed increased order, discrepancies were noted. SAXS indicated intensified short-range correlations, while POM showed the formation of local nematic domains or tactoids. We propose three distinct ordering regimes to reconcile these observations: large-scale nematic order, enhanced short-range order, and short-range clusters. We attributed the temperature-enhanced ordering phenomenon to lateral interactions between the BCSCs, annealing, and memory effects during melting and crystallization.

## Introduction

Thin platelet colloids in solution exhibit a rich phase behavior that has attracted considerable attention in both scientific researches and industrial applications.^1−6^ The driving force behind this phase transition, as explained by Onsager,^7^ is primarily entropy, where the gain in translational entropy outweighs the loss of the orientational entropy in the ordered phase. Theoretical studies and simulations predict that for monodisperse platelets with a sufficiently large aspect ratio, there is an I–N–C (isotropic, nematic, and columnar) phase transition as the volume fraction increases.^7−11^ Experimentally, various liquid crystalline phases, including the nematic phase (N),^12−21^ columnar phase (C),^15,16,22^ lamellar phase (L),^23−26^ or smectic phase (S),^25^ have been reported for different plate-like systems. Current research on the plate-like colloidal systems, particularly mineral nanoparticles, is focused on providing quantitative descriptions of the effects of polydispersity, gravity, gelation, and external magnetic field on the control of phase behavior and applications.^1,4^

The I–N transition of hard platelet suspensions is solely entropy-driven, depending only on the volume fraction and exhibiting no temperature dependence. However, in real colloid systems, soft interactions between the particles are often present. These interactions introduce enthalpic contributions to the phase transitions, making them temperature-dependent. For example, charged colloids may show temperature-dependent phase behavior if the dielectric constant of the solvent changes faster than linearly with inverse temperature, which is often the case for polar liquids, such as water.^27,28^ Toyotama and Yamanaka studied the influence of temperature on the phase behavior of a dilute aqueous solution of charged colloids. With an increase in temperature, the low-charge, low-salt colloids show a freezing transition, whereas the high-charge, high-salt colloids undergo a melting transition. Both transitions are thermoreversible and can be rationalized by the decrease in the permittivity of water at a high temperature.^27^ Li et al. studied the temperature-dependent I–N transition of charged nanoplates in aqueous solution. They found that the N phase melts by increasing temperature, and the I–N coexistence region broadens due to the polydispersity of the colloidal plates.^28^ Dogic et al. studied the temperature-dependent isotropic–cholesteric phase transition in suspensions of virus filamentous bacteriophage *fd*.^29^ They found that the cholesteric pitch shows a rapid unwinding with increasing temperature, and this process is reversible. The temperature dependence of cholesteric pitch is explained as the structural symmetry transitions or changing of surface charge.^29^ These studies and many others have demonstrated the profound impact of temperature on the I–N transition in plate-like colloids. Understanding this relationship is essential for designing and controlling the behavior of these systems for various applications.^30,31^ While these studies have shown the importance of temperature, studies in this direction are still rare.

In our previous works, we have reported a method to prepare a new type of plate-like system from crystallization of a semicrystalline block copolymer, polystyrene-*block*-poly(l-lactide) (PS-*b*-PLLA).^32−35^ These block copolymer single crystals (BCSC) are unique plate-like colloids in the sense that they have a sandwich structure with a uniform thickness and a controllable distribution of the lateral size. In solution, they are stabilized by the steric effect from the tethered PS block on the crystalline core.^36^ Interestingly, in nonpolar solvents such as *p*-xylene, the PLLA crystalline core exhibits lateral attraction, which causes the I–N transition covering a broad range of concentration and size. Due to the lateral attraction, the I–N transition occurs at a very low volume fraction (<0.2%), which is at least 1 order of magnitude lower than the theoretical prediction (2–7%). The solution structures of the crystals and the N phase were further characterized using ultrasmall-angle X-ray scattering (USAXS/SAXS).^35^ Scattering of the individual crystal in solution can be simplified using a simple disc model with a core layer of 10 nm. The isotropic phase could be a coexistence of single crystals and stacked multiple-layered clusters. The face-to-face spacing, *d*, in the N phase is about 75–90 nm, which increases slightly with increasing size of crystals. Due to the lateral attraction between the crystals, it is possible to form even larger crystal sheets and lamellar domains.^35^

Despite the significant progress made in previous works,^32−35^ little is known about the temperature-dependent phase behavior of this system. It is known that BCSCs could anneal upon heating, although thickening is not possible due to the presence of the PLLA side methyl groups. Annealing of BCSCs could make crystals more regular in shape and size. Additionally, increasing temperature will increase the thermal energy of individual particles, which can reduce the effect of gravity, as gravity often plays a crucial role in the phase behavior of anisotropic particle suspensions.^32,33^ In this work, we employed real-time SAXS/wide-angle X-ray scattering (WAXS) techniques to monitor the phase behavior of BCSC solutions in the isotropic and nematic phases during heating and self-seeding and quenching procedures. This approach provided valuable insights into the liquid crystalline phase behaviors of these plate-like colloidal particles. Understanding and controlling these phases are crucial for the design and development of advanced functional materials with unique properties.

## Experimental Section

### Materials and Sample Preparation

The PS-*b*-PLLA was purchased from Polymer Source, Inc. and used as received. The number-average molecular weight (*M*_n_) and polydispersity were measured to be 45,300 (*M*_n_ PS, 21,000; *M*_n_ PLLA, 24,300) and 1.1 by gel permeation chromatography (GPC), respectively. According to the producer (Polymer Source), the PS-*b*-PLLA was prepared by living anionic polymerization in sequential addition of styrene followed by lactide monomer or by taking the OH end-functionalized polystyrene and using a coordination polymerization process. The PS block was atactic with a *T*_g_ = 97 °C and the PLLA block was exclusively l-form to minimize the influence of the optical isomer ratio on polymer property.^37^*p*-Xylene (ρ = 0.857 mg/mL) was obtained from Sinopharm Chemical Reagent Co., Ltd. and used as received.

Single crystals of PS-*b*-PLLA were prepared according to the self-seeding technique reported previously.^32,33^ In brief, a 0.3 mg/mL solution of PS-*b*-PLLA in *p*-xylene was first heated to 130 °C for 30 min to completely dissolve, and then the solution was quenched and stored at 4 °C for 36 h to generate the supersaturated solution. Next, the supersaturated solution was heated to different seeding temperatures for 30 min to form BCSCs with different sizes. Subsequently, the solutions were crystallized at 94 °C in an oil bath for 12 h. For seeding temperatures of 104 and 105 °C, the resulting hexagonal single crystals had an average effective diameter ⟨*D*⟩ of 550 and 1000 nm, respectively, with a standard deviation of 10%. The higher self-seeding temperatures led to a larger average size of the single crystals. The crystals were further collected using membrane filters with a pore size of 0.25 or 0.45 μm (Millipore Corporation). The membranes with crystals were washed three times with *p*-xylene to remove the uncrystallized molecules, and then the crystals were redispersed in *p*-xylene for further use. The suspensions were filled in quartz capillaries for phase behavior and structure characterization by SAXS.

### Characterization Methods

The phase behavior of the suspensions was characterized by using crossed polarizers with a charge-coupled device (CCD) camera at room temperature (RT). This method was widely used to characterize the liquid crystalline phase behavior of plate-like colloidal systems.^12,14,38^ It provides not only solid evidence of the liquid crystalline structure but also the macroscopic orientation of the phase domains. More details can be found in a previous work.^32^

Transmission electronic microscopy (TEM) was performed on a JEM 1400 TEM with an accelerating voltage of 120 kV. The TEM was equipped with a charge-coupled device camera that was used to take micrographs. The samples were prepared by dropping about 10 μL of the crystallizing solution directly onto a carbon-coated copper TEM grid before filtration and allowing the solvent to evaporate under ambient conditions. Tapping mode atomic force microscopy (AFM) was conducted under ambient conditions using an SPA-300HV with an SPI3800N controller (Seiko Instruments Industry Co., Ltd., Japan). Etched Si tips with a resonance frequency of ∼70 kHz and a spring constant of ∼2 N/m were used, and the scanning rate was 1.0 Hz. Each scan line contains 256 pixels, and the whole image is composed of 256 scan lines. A drop of crystallizing solution was deposited on a piece of silicon wafer and spin-coated at 2000 rpm for 30 s, and then the topology of the single crystals was determined.

Small-angle X-ray scattering (SAXS) experiments were carried out on the biological small-angle X-ray scattering (BioSAXS) beamline (BL19U2) at the Shanghai Synchrotron Radiation Facility (SSRF). The X-ray wavelength was 1.033 Å. The calibrated distance from the sample to the detector was 5080 mm. Sample solutions in 1.5 mm quartz capillaries with a wall thickness of 0.01 mm for phase behavior were used directly for the SAXS measurements. The data sets were reduced by subtracting the scattering of a pure solvent as a background. Further details on beamline, *q*-resolution, calibration, and data reduction can be found in refs (38,39). One-dimensional SAXS profiles were converted from the two-dimensional (2D) patterns using the Fit-2D program.

**Scheme 1:**
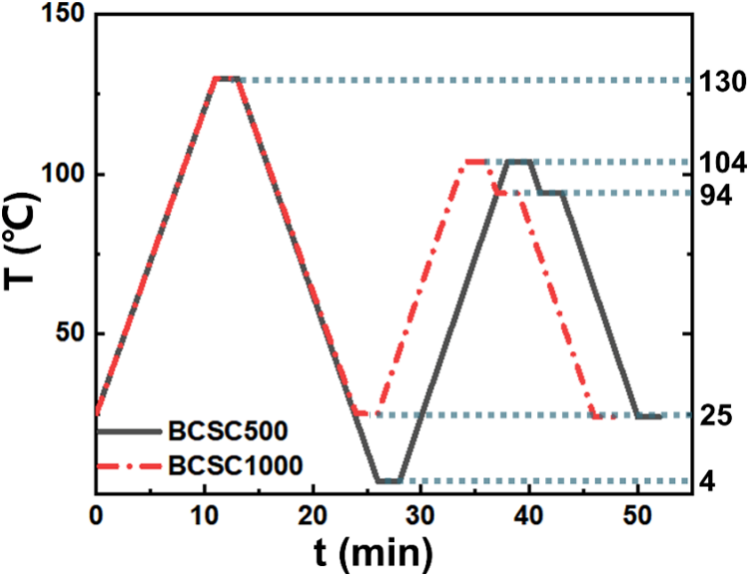
Thermal History of Samples during SAXS/WAXS Measurements with a Scan Rate of 10 °C/min The first quench for BCSC1000 was interrupted at 25 °C due to the emptying of the liquid nitrogen tank.

BCSC500 in the isotropic phase with a concentration of 48 mg/mL and BCSC1000 in the N phase with an initial concentration of 33 mg/mL were filled into a capillary for the SAXS/WAXS measurements. The measurement procedure followed Scheme 1. Due to the emptying of the liquid nitrogen tank, the BCSC1000 sample quenching was interrupted at 25 °C. This difference in quench depth may have compromised the stability of the resulting crystals.

Polarization optical microscopy (POM) measurements were conducted using an Axio Imager A2m microscope (Carl Zeiss, Germany) equipped with a temperature-controlled stage. To ensure consistency with SAXS measurements, the same sample solutions in capillaries (1.1 mm diameter) were placed on top of the stage. Images at different temperatures were collected, and representative images capturing the phase changes in response to temperature were presented.

## Results and Discussion

### Structure Characterization of BCSCs

**Figure 1:**
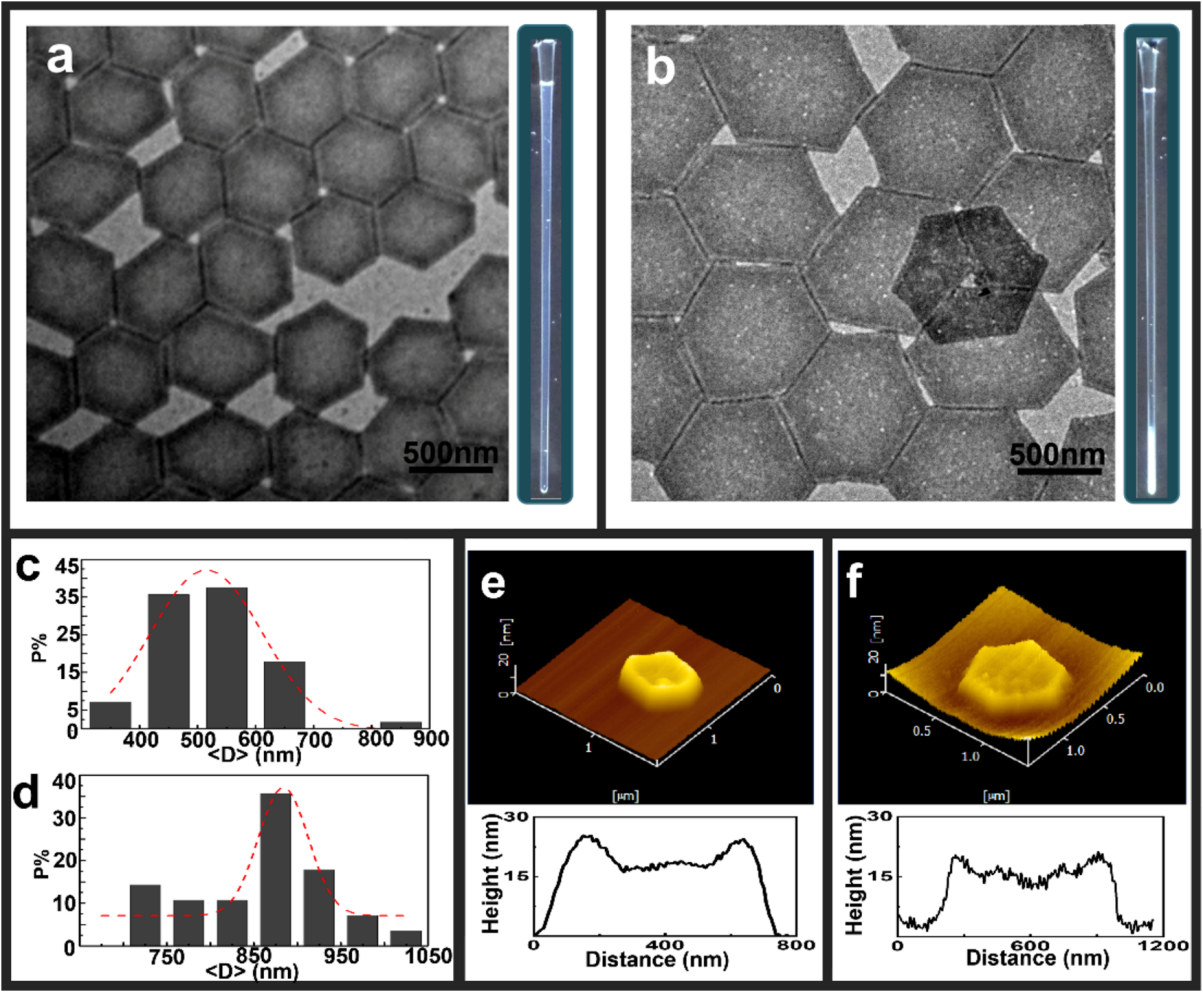
BCSC samples used in this work: TEM images of BCSCs of different sizes prepared by the self-seeding technique, and the photographs of capillaries for (a) BCSC500 (48 mg/mL) and (b) BCSC1000 (33 mg/mL). The scale bar in TEM is 500 nm. The statistical analysis of size distribution gives the mean size ⟨*D*⟩ of (c) 525 ± 95 nm and (d) 883 ± 83 nm for BCSC500 and BCSC1000, respectively. Representative AFM height images of BCSC500 (e) and BCSC1000 (f) show an average height of 18.0 ± 0.5 nm.

BCSCs of different lateral sizes have been prepared using a self-seeding technique. Previous works^32,34^ on the control parameters of crystallization have shown that the seeding temperature controls the number of crystals; thus, the final size and crystallization temperature, *T*_c_, control the morphology of the crystals, and a transition from lozenge shape to truncated lozenge crystals occurs with increasing *T*_c_.^34^ The lozenge shape crystals used in previous works were obtained at *T*_c_ of 75 °C.^32,33^ In this work, to reduce the geometric anisotropy of the crystals, truncated lozenge crystals were prepared at *T*_c_ of 94 °C. Typical TEM images of the truncated lozenge crystals are presented in Figure 1a,b together with the statistical analysis of size distribution. The crystals are nearly hexagonal, and their mean size as well as size distribution increase with the seeding temperature. The equivalent diameter ⟨*D*⟩, which is the diameter of a circle with the same area of the hexagon, is used to describe the size of crystals from different seeding temperatures. The ⟨*D*⟩ values of BCSC500 and BCSC1000 are 525 ± 95 and 883 ± 83 nm, respectively. The average overall thickness ⟨*L*⟩ of BCSC is 18.0 ± 0.5 nm determined by AFM, which is a constant for all crystals as it only depends on the crystallization temperature. The thickness of the middle crystalline PLLA layer is 9.0 nm, calculated using the following equation^32,40^
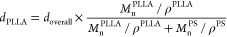
1where *d*_PLLA_ and *d*_overall_ are the thickness of PLLA layer and the overall thickness of the BCSC, respectively; *M*_n_^PLLA^ and *M*_n_^PS^ are the number-average molecular weights of PLLA and PS; ρ^PLLA^ = 1.28 g/cm^3^ and ρ^PS^ = 1.05 g/cm^3^ are the density of crystalline PLLA and amorphous PS, respectively. The PS layer in the dry state is about 4.5 nm. The smallest size of BCSC used here is 525 nm, so the aspect ratio, ⟨*D*⟩/⟨*L*⟩ is larger than 16.7 for all systems used in this work. According to the theoretical prediction,^9,10^ when ⟨*D*⟩/⟨*L*⟩ ≥ 8.3, an I–N transition is expected.

In this work, we focus on two samples: one is BCSC500 with a concentration of 48 mg/mL, and the solution at room temperature is in the isotropic phase (Figure 1a). The other sample is BCSC1000 with an initial crystal concentration of 33 mg/mL (Figure 1b). This suspension undergoes I–N phase transition at room temperature, as shown in the inset of Figure 1b. The concentrations of the I and N phases are 1.4 ± 0.5 and 223 ± 20 mg/mL, respectively. The N phase of the second sample is used for the following temperature-dependent measurements.

### Enhanced Ordering during Annealing—Heating from RT to 130 °C

**Figure 2:**
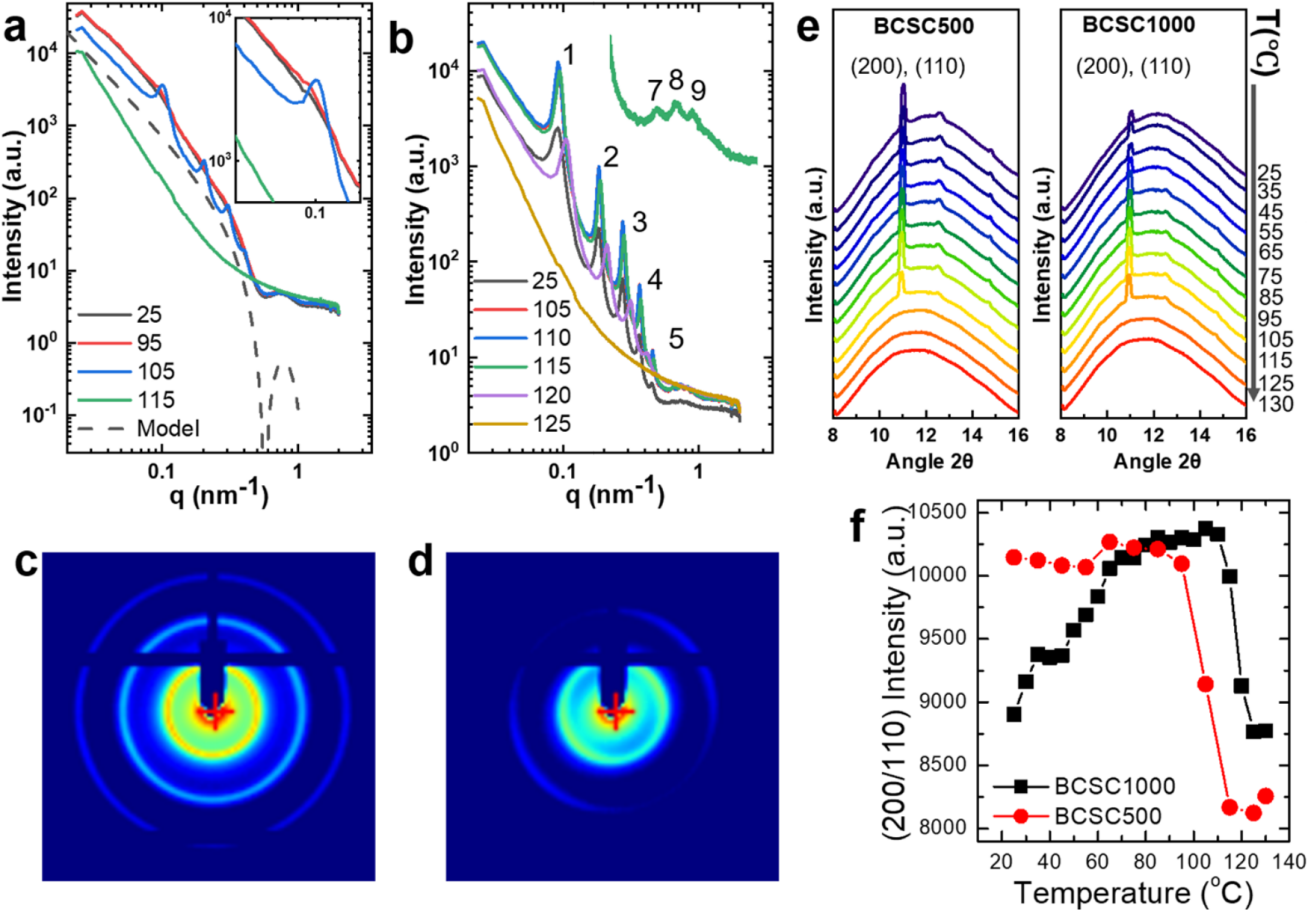
SAXS/WAXS during heating from 25 to 130 °C for BCSC500 in the isotropic phase and for BCSC1000 in the N phase: (a) selected SAXS profiles for BCSC500, dashed line corresponds to the form factor of a single layer disc model; (b) SAXS for BCSC1000 (for comparison, plots with the curves shifted up–down are shown in Figure S2), (c, d) 2D SAXS patterns at 115 and 120 °C, anisotropic SAXS pattern at 120 °C indicates the formation of a long-range order in N phase. (e) WAXS profiles around the (200) and (110) peaks for both systems. (f) Plots of the intensity of (200) and (110) peaks for both systems during heating; a strong annealing effect is observed for BCSC1000.

Figure 2a,b presents the SAXS profiles for the two samples during heating from room temperature to 130 °C. At room temperature, the BCSC500 sample, with a concentration of 48 mg/mL, is in the isotropic phase, and the face-to-face correlation is not visible. In a previous work,^35^ a comparable system (BCSC500) at 10 mg/mL displayed this ordering without undergoing an I–N transition. In this study, the mean size of the BCSC is slightly smaller than the previous one. This difference highlights the influence of crystal mean size on the formation of plate-clusters or the N phase, particularly for the small crystals with a ⟨*D*⟩/⟨*L*⟩ ratio close to the theoretical I–N transition. Furthermore, the polydispersity of the crystal size may also contribute to the different phase behavior.

Scattering of the BCSC single crystal was described using a simple single layer disc model to describe the individual crystals, which is included in Figure 2a for comparison.^35^ In Figure 2, the features in the experimental SAXS curve above *q* = 0.2 nm^–1^ reflect the form factor of the individual crystal, and the minimum located at *q* = 0.58 nm^–1^ corresponds to the first scattering minimum of the form factor of a disc. The primary peak at *q* = 0.10 nm^–1^ corresponds to the interlayer correlation. For disc-like particles in solution, the scattering intensity in the intermediate *q* range can be approximated as . Here, *h* is the thickness of discs. Thus, the thickness can be determined using a modified Guinier plot: ln[*I*(*q*)*q*^2^] vs *q*^2^. This analysis was applied to BCSC500 at 25 °C (Figure S1), yielding a mean thickness of 14.3 ± 1.5 nm. This value is smaller than that determined by AFM in the dry state (Figure 1e,f), which may be due to the low contrast in SAXS for the tethered PS blocks in *p*-xylene, as discussed in the previous work.^35^

During heating from 25 to 130 °C, the SAXS profiles of the BCSC500 sample (Figure 2a) remain unchanged in the entire *q* range until 95 °C. At 95 °C, a subtle bump appears near the first correlation peak, but no clear signs of ordering or correlation peaks are detectable. However, at 105 °C, sharp and intense correlation peaks at *q* = 0.10, 0.20, 0.30, and 0.40 nm^–1^ (in a 1:2:3:4 ratio) emerge, extending up to the fourth order, indicating the formation of an ordered structure. Upon reaching 115 °C, the BCSCs fully melt and the ordering disappears. It is important to note that due to the heating rate of 10 °C/min and the SAXS acquisition interval of 1 exposure per minute, SAXS profiles were only collected every 10 °C, preventing the visualization of intermediate states. Given the molecular weight of the PS block (21 kDa), its fully extended length is 31 nm. When combined with the crystalline core thickness of 9 nm, the face-to-face spacing without overlapping PS blocks is approximately 71 nm. However, grafted PS blocks on crystals in solution typically have a mushroom-like conformation, resulting in a much shorter grafted PS layer. The observed face-to-face spacing in this work, ranging from 63 to 70 nm, suggests that the crystals do not have direct contact or overlap. This contrasts with polyethylene crystals formed in solution, where SAXS/small-angle neutron scattering (SANS) studies reveal correlation peaks at 0.25 and 0.50 nm^–1^, corresponding to the first- and second-order correlations between lamellae, indicating close packing with a spacing of around 25 nm.^41,42^

In the N phase of BCSC1000 (Figure 2b), pronounced correlation extends up to the fifth order of the correlation peak at room temperature. SAXS results at different temperatures clearly demonstrate the enhancement of ordering upon heating. The correlation peak sharpens significantly, and up to the ninth order of correlation peaks are observable at 115 °C. The bump in the high *q* (>0.5 nm^–1^) range encompasses the seventh–ninth peaks, while the sixth-order peak aligns with the form factor scattering minimum (*q* = 0.58 nm^–1^) and becomes invisible. This enhanced ordering may lead to the formation of lamellar phases or domains within the N phase, as discussed in the previous work.^35^ Alongside the intensified peak intensity, the peak position also shifts toward higher *q* values with increasing temperature. The first-order correlation peak at 25–110 °C is located at *q* = 0.091 nm^–1^, shifting to 0.094 nm^–1^ at 115 °C and further to 0.104 nm^–1^ at 120 °C. This shift of the correlation peak to higher *q* values indicates a reduction of the interlamellar spacing. This reduced spacing can be attributed to the melting of crystals. In particular, at 120 °C, the overall scattering intensity decreases compared to previous temperatures, suggesting that the crystals have partially melted. Melting of crystals reduces the mean size of the crystals, which is primarily responsible for the shift of the peak to higher *q* values. Another possible contribution to the change in spacing is the conformation change of the tethered PS chains. However, in a good solvent *p*-xylene, tethered PS chains will generally undergo a minimal change in conformation upon heating. Both increased thermal energy of polymer chain and a slightly better solvation may contribute to a small expansion, which would lead to a slight increase of spacing.

Figure 2c,d presents the two-dimensional SAXS patterns for BCSC1000 at 115 and 120 °C. The elliptical SAXS pattern at 120 °C clearly reveals long-range orientational correlations within the N phase. Previous studies have demonstrated that SAXS 2D patterns for BCSC2000 and BCSC4000 exhibit elliptical shapes, while smaller samples exhibit isotropic SAXS 2D patterns.^35^ The orientation of the SAXS 2D pattern may vary depending on the sample position, as has been observed in the N phase of other plate-like systems.^23,43^ The anisotropic SAXS patterns observed for high-concentration and larger crystals indicate the formation of a long-range order within the N phase.^44^

Figure 2e,f presents the WAXS results during heating for both systems. For BCSC500, the WAXS profiles exhibit a sharp Bragg peak before 105 °C, which is attributed to the diffraction from the (200) and (110) lattice planes of the α′- and α-forms of PLLA.^45−47^ The peak intensity does not change significantly with increasing temperature. At 105 °C, the peak intensity decreases and the crystals eventually melt at 115 °C. Conversely, for BCSC1000, the Bragg peaks become sharper during heating and the intensity increases before 115 °C, with even higher-order peaks becoming visible. The intensity eventually decreases and the crystals melt at 125 °C. Figure 2f plots the intensity of the (200) and (110) Bragg peaks as a function of temperature for both systems. For BCSC500 in the I phase during heating, the Bragg peak maintains a constant intensity before 95 °C and then rapidly decreases above that temperature until melting. In contrast, BCSC1000 in the N phase exhibits clear annealing behavior: the Bragg peak intensity quickly increases after 45–70 °C, followed by a slower increase until 110 °C before a sudden decrease with melting. It is worth noting that despite the annealing effect observed for BCSC1000, the crystal thickness remains unchanged, as evidenced by the lack of observable changes at the scattering minimum at *q* = 0.58 nm^–1^. This is consistent with the known fact that PLLA crystals cannot thicken through annealing due to the presence of side methyl groups, and the high thermodynamic stability of crystals can be achieved only by recrystallization.^34^

### Quenching from 130 °C

**Figure 3:**
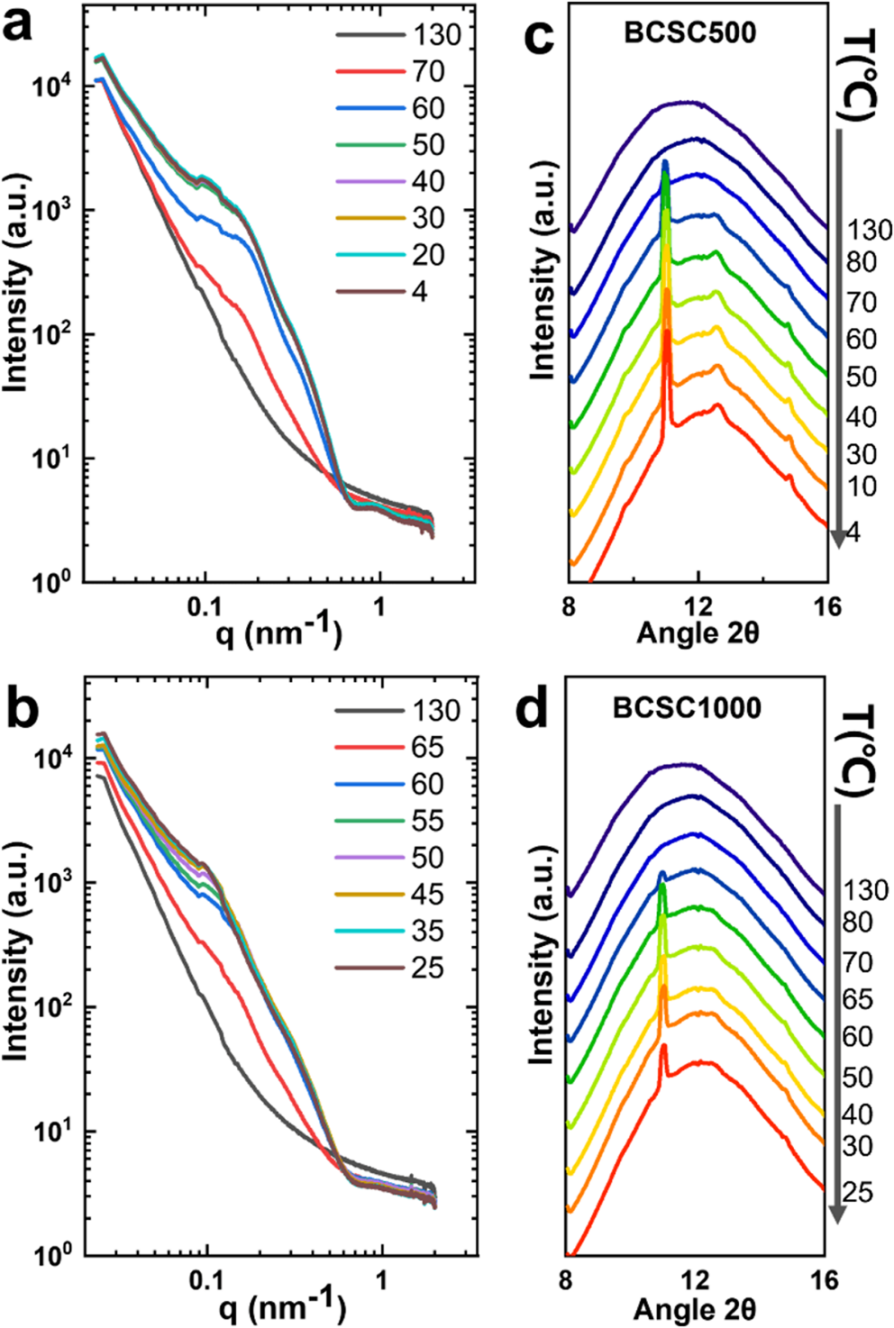
SAXS/WAXS results during quenching from 130 °C for both systems (a, b). Due to the data collection difference, for BCSC500, the SAXS signal appears at 70 °C whereas for BCSC1000, it appears at 65 °C. The corresponding WAXS profiles are consistent with the SAXS results (c, d).

Figure 3 presents the SAXS/WAXS results for both systems during the quenching. SAXS for BCSC1000, the sample was cooled to 25 °C, with data collected every 5 °C. At 65 °C, a broader peak at *q* = 0.1 nm^–1^ develops, and a *q*^–3^ decay follows up to *q* = 0.3 nm^–1^. In the meantime, in the high *q* region, *q* > 0.5 nm^–1^, intensity decreases, and a broad bump emerges. A scattering minimum occurs at *q* = 0.73 nm^–1^, indicating that the crystals formed during the quench are thinner and less perfect with random orientation. For BCSC500, the solution was quenched from 130 to 4 °C with a rate of 10 °C/min and held at 4 °C for 3 min. SAXS signal changes become evident below 70 °C, centered around *q* = 0.2 nm^–1^ and increasing with further decreasing temperature. Below 50 °C, no further changes are observed. The WAXS results in Figure 3b,d corroborate the SAXS observations, with Bragg peaks appearing at 65 °C (BCSC1000) and 70 °C (BCSC500). In both cases, the sharp face-to-face correlation peaks of the plate-like crystals are not visible. This suggests that the thin crystals formed during rapid quenching directly from the melt state are randomly orientated and lack long-range order beyond the crystal size.

### Seeding at 104 °C and Crystal Growth at 94 °C

**Figure 4:**
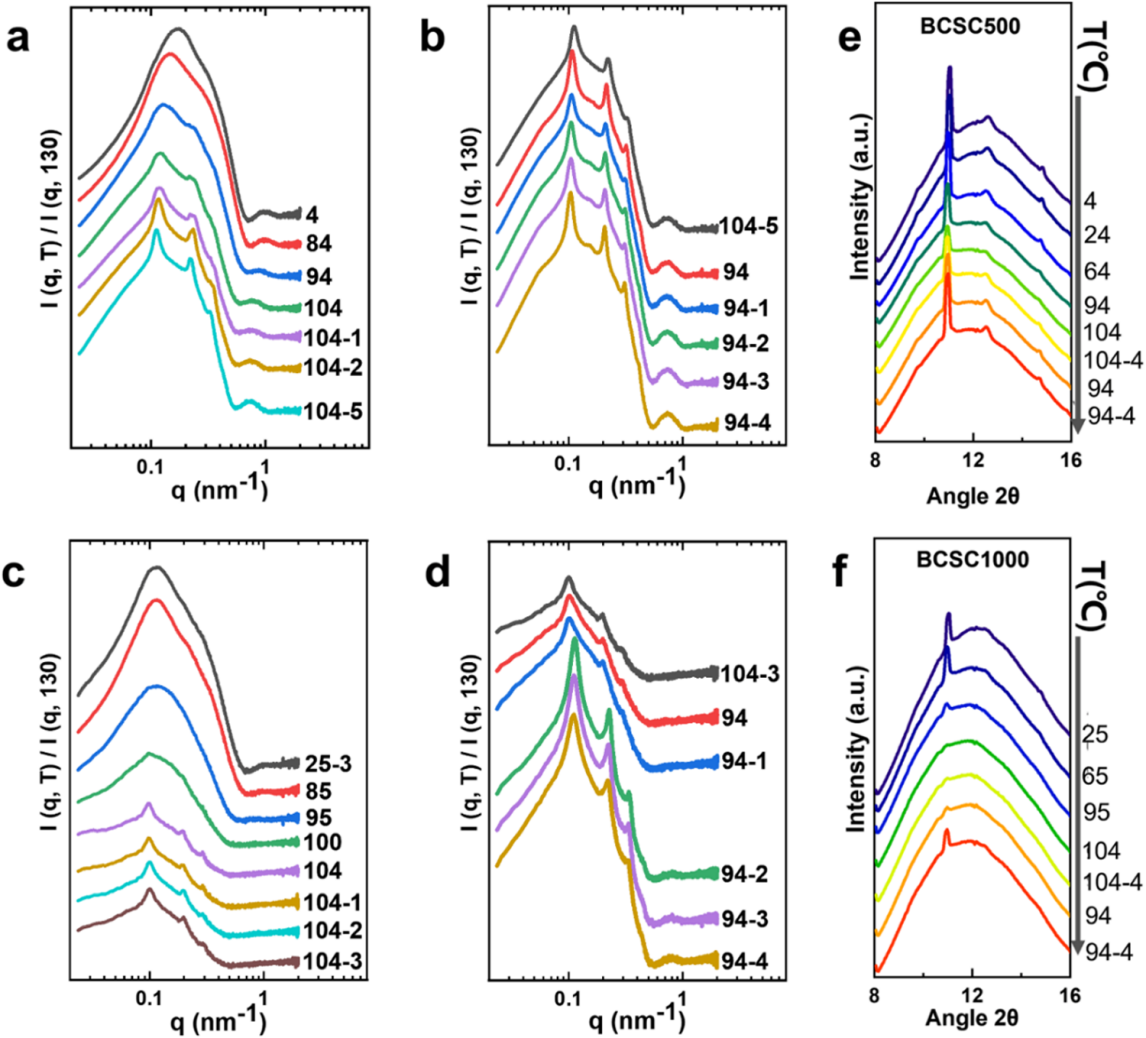
Normalized SAXS profiles for (a, b) BCSC500 and (c, d) BCSC1000 during self-seeding at 104 °C and crystallization at 94 °C (the original intensity plots are in Figures S3 and S4). (e, f) WAXS results for both systems. Note the disappearance of Bragg peaks for BCSC1000 at 104 °C and reappearance at 94 °C.

The samples were further heated from 4 or 25 to 104 °C and held at this temperature for 4 min for self-seeding, followed by crystallization at 94 °C for a few minutes (Figure 4). To clearly observe the subtle changes in the appearance of correlation peaks, the SAXS profiles were normalized using the SAXS curve in the melt state at 130 °C. During heating to 104 °C, the SAXS profiles for both systems exhibit increased intensity in the high *q* region, and the scattering minimum shifts from 0.72 nm^–1^ to a lower value of 0.56 nm^–1^. This shift indicates the formation of significantly thicker crystals as the thinner, less perfect crystals formed during quenching are consumed.

For BCSC500, the first SAXS profile at 104 °C (Figure 4a) exhibits very weak face-to-face correlation peaks. After 1 min, the second curve shows a decrease in overall intensity, indicating some melting of crystals, but the correlation peaks become stronger. The third curve shows an increase in intensity and better correlation, and extending the time to 4 and 5 min results in further intensity increase and a slight shift of the first correlation peak to a lower *q* from 0.117 to 0.111 nm^–1^. Subsequent crystallization at 94 °C (Figure 4b) leads to an overall intensity increase and peak shift to further lower *q* values. After 4 min, the first peak at 0.111 nm^–1^ shifts rapidly to 0.102 nm^–1^, clearly indicating the growth of crystals in size and the increase of the interlamellar spacing. In the region of *q* > 0.5 nm^–1^, the broad bump remains relatively unchanged in both position and intensity, but the minimum at *q* = 0.56 nm^–1^ deepens. This observation suggests that the crystal thickness does not change significantly as the position of the minimum is closely related to the crystal thickness. WAXS results for BCSC500 (Figure 4e) reveal that the Bragg peak retains its presence with reduced intensity as the sample is heated from 4 to 104 °C. Upon crystallization at 94 °C, the Bragg peak regains intensity and high-order peaks become visible, confirming the formation of larger and more ordered crystals.

For BCSC1000, as the sample is heated from 25 to 104 °C (Figure 4c), the scattering intensity at *q* < 0.5 nm^–1^ decreases significantly due to the melting of most crystals. The high *q* intensity increases, but the bump is not as well-developed as that of the BCSC500 system. This is because of the higher quenching temperature (25 °C) resulting in a lower degree of crystallinity. However, after seeding at 104 °C for 4 min, a first correlation peak at about *q* = 0.100 nm^–1^ and second-order peak become visible, indicating the formation of nematic ordering. Subsequent crystallization at 94 °C leads to an increase in the overall intensity (*q* < 0.5 nm^–1^) but the peak position remains unchanged in the first 2 min. At 3 min, a strong correlation peak at *q* = 0.113 nm^–1^ appears and dominates, and judging from the second-order peak, the previous peak at 104 °C has disappeared. After 5 min, the peak shifts slightly to *q* = 0.111 nm^–1^. Also, the intensity at *q* > 0.5 nm^–1^ and the position of the minimum remain unchanged, indicating that the final crystals are of similar thickness. This suggests that the self-seeding step at 104 °C promoted the growth of thicker crystals, but the crystallization at 94 °C further refined the crystals and led to the emergence of stronger correlation peaks. WAXS data for BCSC1000 (Figure 4f) reveal that the Bragg peak disappears above 100 °C during heating. After 4 min at 104 °C, no Bragg peaks are visible. However, upon cooling to 94 °C for crystallization, the Bragg peak reappears and grows in intensity with time. This different behavior from BCSC500 may be due to the higher quenching temperature in the previous step for BCSC1000 (25 °C), resulting in a lower degree of crystallinity.

One interesting aspect of BCSC1000 is that while the WAXS Bragg peaks disappear at 104 °C (Figure 4f), the SAXS signal begins to exhibit a face-to-face correlation (Figure 4c). This behavior can be attributed to the memory effect in polymer crystallization and melting^48,49^ where the melted copolymer crystals do not fully dissolve in solution. Instead, they retain their global shape with the crystalline core transitioning to a molten state. As a result, no Bragg peaks are observed in WAXS, but weak correlation peaks are visible in SAXS. This hypothesis also accounts for the shift in correlation peaks observed in SAXS for BCSC1000. As the crystals formed during quenching primarily melt at 104 °C, the weak correlation peaks originate from the melted crystals, as explained earlier. Consequently, their positions and intensities remain unchanged over time. When crystallization resumes at 94 °C, there are insufficient seeds left, and new nucleation occurs within the melted crystals, ultimately dominating the final crystallization process. This leads to the emergence of a new strong correlation peak.

### Final Cooling Down to 25 °C

**Figure 5:**
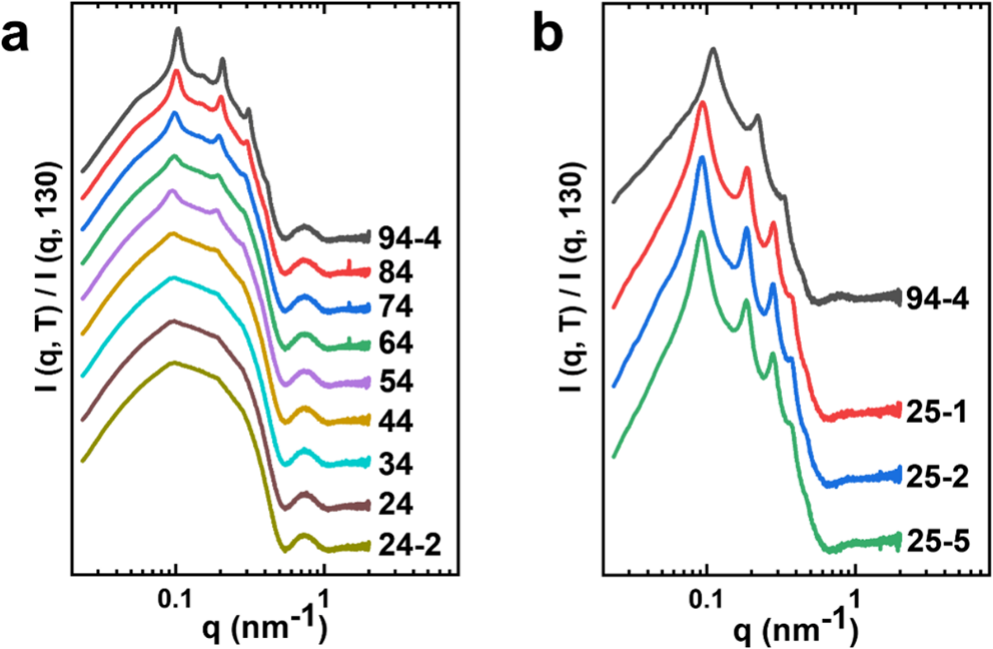
Normalized SAXS profiles during the final quench from crystallization temperature of 94 to 25 °C for (a) BCSC500 and (b) BCSC1000 (the original intensity plots are in Figure S5). Note the correlation peaks gradually disappear for BSCS500 but remain for BCSC1000 during the final cooling.

Following crystallization at 94 °C for 5 min, the solution was cooled to room temperature. Remarkably, upon cooling, the order disappeared gradually for BCSC500 in SAXS (Figure 5a). Specifically, during cooling, the first correlation peak shifts slightly from *q* = 0.102 nm^–1^ at 94 °C to a lower value of 0.094 nm^–1^ at 64 °C. In the meantime, the peak intensity decreases and peaks broaden. Below 44 °C, the ordering appears lost based on the second-order peak. In contrast, the correlation peaks (thus ordering) for BCSC1000 remained intact after cooling (Figure 5b). The first-order peak shifts from *q* = 0.106 to 0.094 nm^–1^ at 25 °C and gains in intensity. As expected, WAXS for both systems gives clear Bragg peaks after cooling (Figure S6). These observations suggest that the ordering of BCSC500 is more susceptible to thermal fluctuations, leading to a gradual loss of long-range order during cooling. In contrast, BCSC1000 in the N phase exhibits greater stability in its ordering, maintaining its structure even at lower temperatures.

### Phase Behavior Observed Using POM

SAXS measurements presented above indicate that the plate–plate correlation order increases with temperatures. This finding prompted us to investigate macroscopic nematic order using POM. Figure S7 shows photographs under a polarizer for sample capillaries of BCSC500 in the I phase and BCSC1000 in the N phase. While BCSC500 exhibits uniform darkness in the I phase, BCSC1000 displays characteristic Schlieren textures in the N phase.

**Figure 6:**
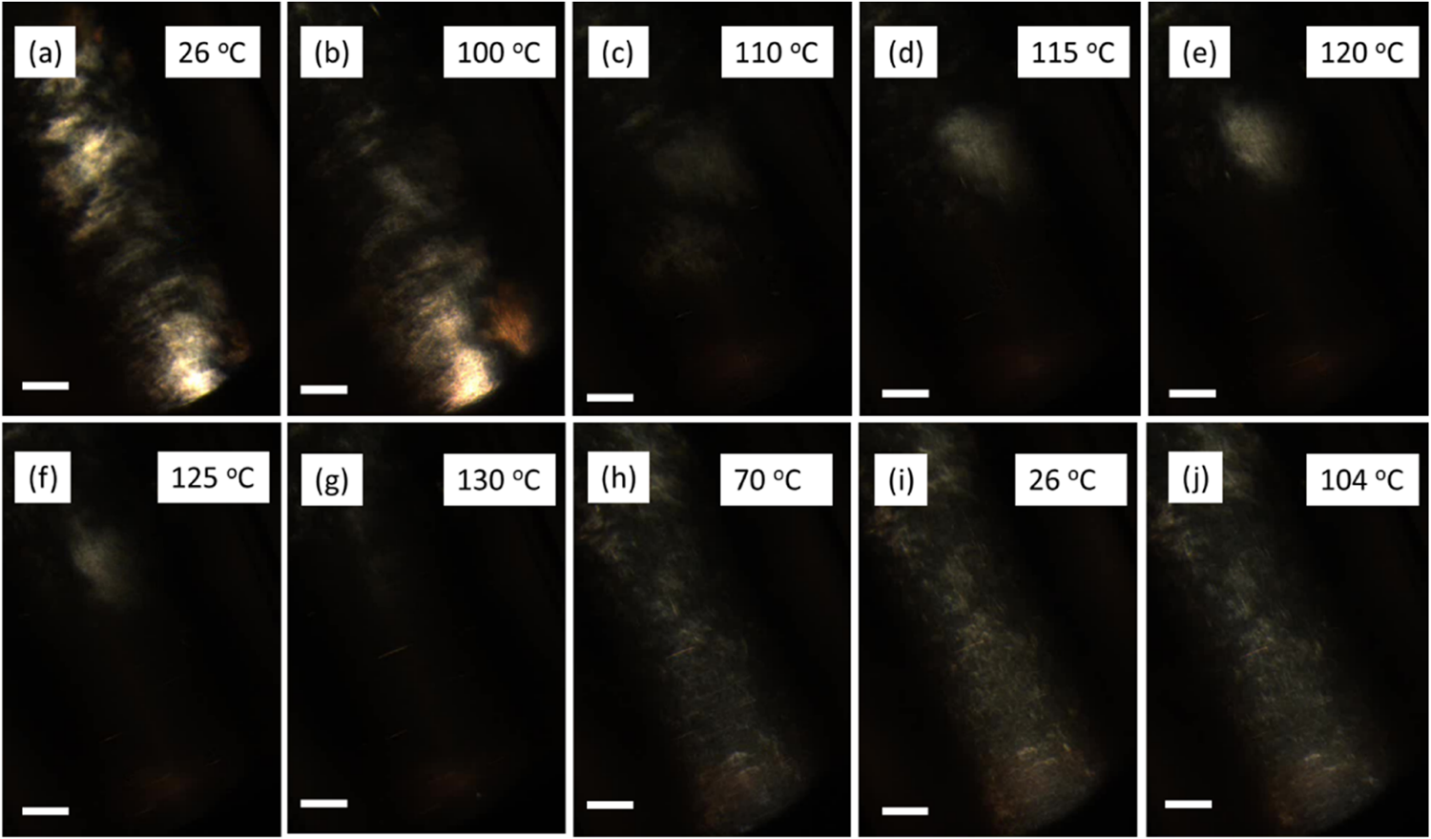
POM micrographs of BCSC1000 in the N phase during (a–g) heating, (h, i) cooling, and (j) then reheating to 104 °C with a constant scan rate of 10 °C/min. Scale bar is 100 μm.

Figure 6 presents a series of POM images of BCSC1000 during a heating cycle at a rate of 10 °C/min. At room temperature (Figure 6a), the capillary shows typical Schlieren textures indicative of nematic order. As the temperature increases to 100 °C (Figure 6b), the texture brightness diminishes, suggesting a decrease in nematic order, and at 110 °C (Figure 6c), the texture nearly vanishes, indicating a transition to an isotropic phase. Concurrently, temperature gradients induce material flow toward the top of the capillary. However, a distinct region with finer Schlieren texture emerges in the middle of the view around 115 °C (Figure 6d), grows brighter at 120 °C (Figure 6e), and then decreases at 125 °C (Figure 6f) until all textures vanish at 130 °C (Figure 6g). Upon quenching from 130 °C to room temperature, Schlieren textures reappear below 100 °C, becoming brighter with decreasing temperature, suggesting both recrystallization of PLLA and the reformation of the nematic order. Below 70 °C (Figure 6h), no significant changes are observed. The resulting texture (Figure 6i) differs from the initial 26 °C texture, exhibiting a lower overall brightness. Further heating to 104 °C (Figure 6j) results in a slight brightness reduction without altering the overall texture. Similarly, at 94 °C, the texture remains unchanged but the brightness increases slightly.

**Figure 7:**
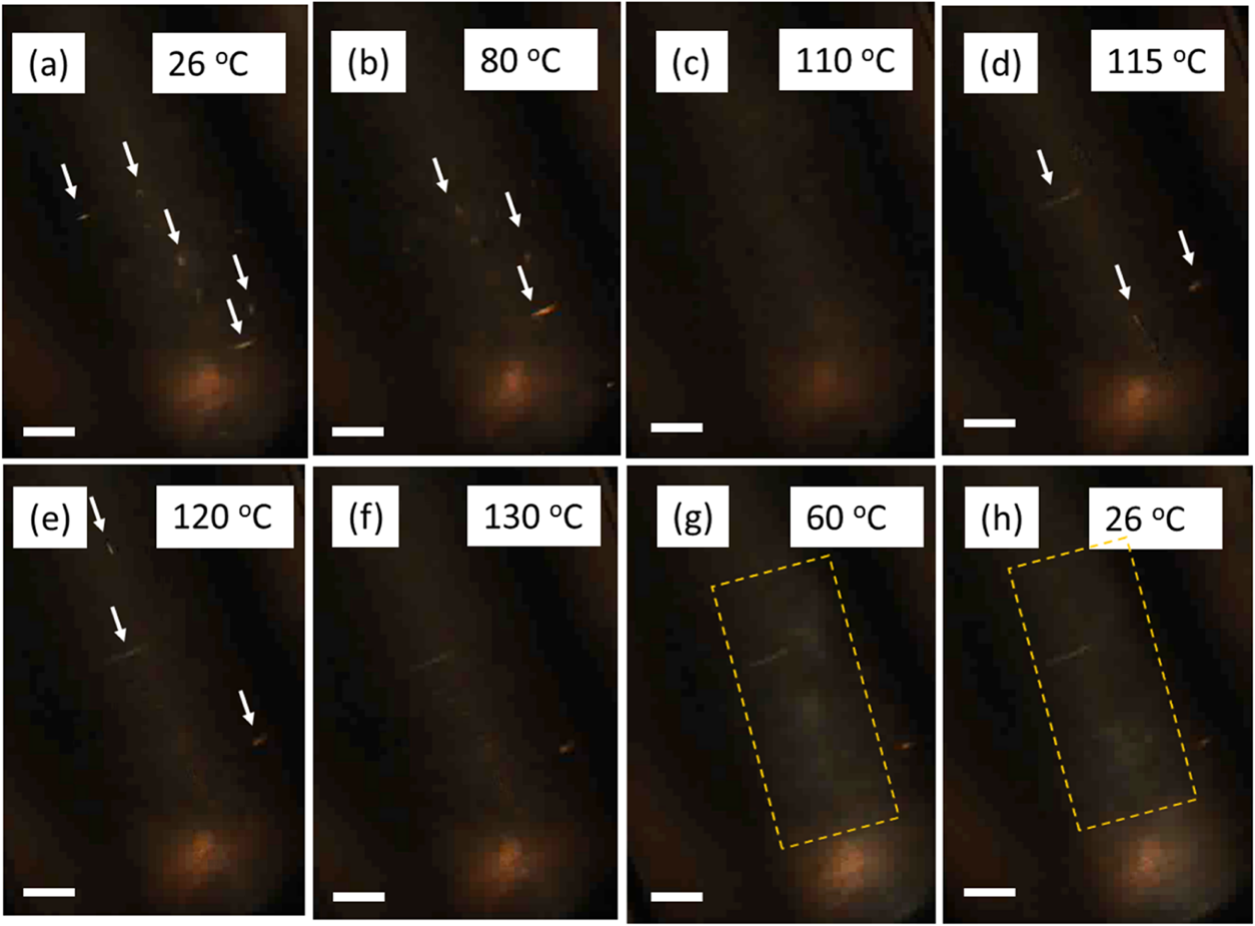
POM micrographs of BCSC500 in the I phase during (a–f) heating and (g, h) cooling with a constant scan rate of 10 °C/min. The bright spot at the bottom of the images is caused by the end of the capillary. Scale bar is 100 μm.

Figure 7 presents a series of POM images for BCSC500 in the isotropic phase during the heating and cooling cycles. To enhance visibility, image contrast has been adjusted. At room temperature (Figure 7a), a few bright rod- or biconvex-shaped objects are visible as indicated by white arrows. Despite the solution being in the I phase, the high concentration of BCSC500 particles likely facilitates the formation of tactoids, precursors to the nematic phase. These tactoids can move with the flow during heating, gradually losing brightness and dissolving completely at 110 °C (Figure 7b,c). However, starting at 115 °C (Figure 7d), a noticeable overall brightness increase along the capillary center occurs, accompanied by the emergence of new tactoids. The brightness and the number of tactoids decrease with further increase in temperature to 130 °C (Figure 7e,f). This reappearance of tactoids aligns with SAXS measurements (Figure 2a) where strong correlation peaks appear before melting. However, given the number of tactoids observed in POM, the SAXS observation may be because of the random alignment of some tactoids within the X-ray beam. During cooling, tactoids reappear around 100 °C, and below 70 °C (Figure 7g,h), the overall brightness increases as indicated in the dashed rectangular box, suggesting the recrystallization of PLLA, but not the nematic order.

### Discussion on the Annealing Effect and Thermal Enhanced Ordering

Here, we first summarize the main observations of this study: in general, we have observed enhanced ordering at higher temperatures for our BCSCs in solutions. For example, the BCSC500 in the isotropic phase at room temperature shows a well-developed order in SAXS during heating at 105 °C, potentially due to the tactoids, as observed by POM, randomly aligning within the X-ray beam. For BCSC1000 in the N phase, the order is enhanced during heating, and up to the ninth order of correlation is visible at 115 °C. After melting at 130 °C, both solutions were quenched, and crystals formed during quenching do not show the order. The samples are further heated to 104 °C for seeding, the order appears again, but for BCSC1000, the correlation is most likely from the molten crystals as Bragg peaks completely disappear in WAXS. Upon subsequent crystallization at 94 °C, the order in both systems is further developed. Interestingly, upon the final cooling to room temperature, the order for BCSC500 is gradually reduced and eventually disappears, whereas the order for BCSC1000 remains. All of these observations indicate that the high temperature favors ordering.

POM observations provide additional insights into the temperature-dependent behavior of these systems. In both cases, we observed enhanced ordering prior to crystal melting, aligning with the SAXS findings. However, some discrepancies exist: first in POM, the overall brightness of the Schlieren texture initially decreases with increasing temperature, whereas in SAXS the correlation peaks increase gradually; Second, despite being in the isotropic phase, BCSC500 exhibits tactoids, precursors to the nematic phase, in POM images.

**Figure 8:**
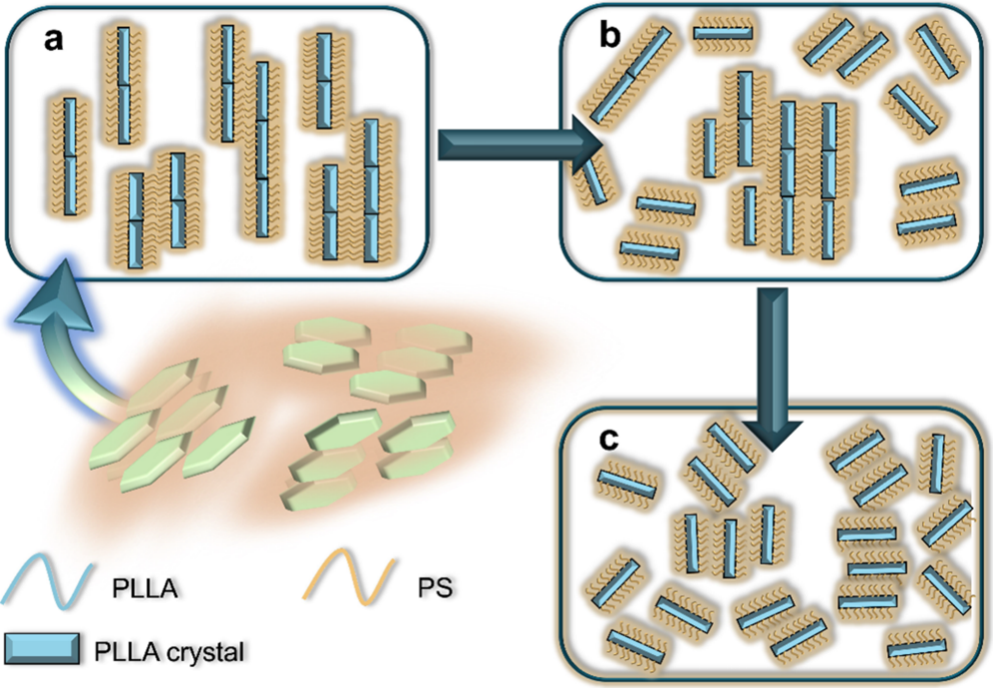
Three orderings formed by the plate-like BCSCs in solution: (a) Large-scale nematic order visible both in SAXS and POM; (b) enhance short-range order, the local area with dashed circles indicates the enhanced nematic domains, visible both in SAXS and POM; and (c) short-range clusters, visible only in SAXS. A schematic illustration of BCSC is shown in the bottom-left panel. For simplicity, the rod-like objects with tethered PS blocks represent plate-like BCSCs.

These differences lead to the first discussion of the length scales of the ordering observed by SAXS and POM. As depicted in Figure 8, three different ordering regimes are observed: (a) Large-scale nematic order: Plates align over a large scale, forming a nematic (N) phase domain visible under POM. Simultaneously, plate-to-plate correlations are detectable by SAXS. (b) Enhanced short-range order: During heating, face-to-face correlations between plates intensify, as indicated by peak shifts to higher *q* values in Figure 2b. However, although long-range alignment is lost in most areas of solution, small nematic domains or tactoids with this short-range order can be visualized under POM, as seen in Figure 6 at 120 °C and the tactoids forming at 115–120 °C in Figure 7. (c) Short-range clusters: Plates stack into clusters with strong face-to-face correlations, but the overall orientation remains random. This type of order is exclusively observable by SAXS.

We now discuss the following question: Why does a high temperature enhance face-to-face short-range ordering? As the temperature increases, the thermal energy of the platelets increases, which normally leads to the melting of the nematic phase. On the other hand, it also makes it easier for the plates to overcome the forces that prevent their alignment. This increased thermal energy allows the platelets to move more freely and align more closely together, leading to the formation of a short-range order. The exact mechanism by which the temperature affects the I–N transition is complex and depends on the specific properties of the platelets and the solvent. However, two main factors may be involved: First, the thermally activated hopping mechanism may play a role:^50,51^ At low temperatures, the platelets are locked into a disordered state due to strong interparticle interactions (e.g., polar attraction between the PLLA crystalline core and gravity). However, as the temperature increases, the thermal energy of the particles increases, allowing them to overcome these interactions and jump to new positions. This increased mobility of the platelets makes it easier for them to find new, more energetically favorable orientations. This process can lead to the spontaneous formation of small clusters of aligned platelets, which can then grow and merge to form a larger nematic phase. Second, the increased thermal motion of the platelets also increases the fluid friction between the platelets and the solvent, generating a hydrodynamic shear effect that can act to align the platelets, effectively pushing them into a more ordered state.^52,53^ The combination of these two mechanisms leads to an increase in the rate at which the I–N transition occurs as the temperature increases.

In our BCSC system, it is known that the crystalline PLLA core causes lateral attractive interactions.^32,33,35^ The polar interaction between the crystalline PLLA core layers of polystyrene-*block*-poly(l-lactide) (PS-*b*-PLLA) block copolymer single crystals in *p*-xylene can enhance the isotropic-to-nematic (I–N) transition. This is because the polar interactions can act as a driving force for nematic ordering by aligning the platelets, forming larger sheets, increasing the ⟨*D*⟩/⟨*L*⟩ ratio and reducing the entropy penalty associated with their alignment. The polar interaction between the PLLA core layers may be due to the formation of hydrogen bonds between the carbonyl groups and the H atoms in the methyl groups of the PLLA chains as reported in the literature.^54,55^ These hydrogen bonds can be disrupted by increasing the temperature, which can lead to destabilization of the nematic phase. However, when the polar attraction is strong, it can significantly reduce the entropy of the platelets; increasing temperature can enhance the I–N transition by weakening these interactions and allowing the platelets to move more freely.

Another important issue in our system is the annealing effects: Annealing provides additional thermal energy that allows the PLLA chains to relax and organize into a more ordered crystalline structure. This improved crystallinity can lead to stronger polar interactions between the PLLA core layers, which, in turn, can stabilize the nematic phase. Annealing can also reduce the number of defects present in the PLLA core layers. These defects can act as nucleation sites for the growth of disordered or partially ordered domains, which can hinder the formation of a stable nematic phase. By reduction of the number of defects, annealing can promote the formation of a more homogeneous and ordered nematic structure.

TEM images in Figure 1 reveal that BCSC500 crystals exhibit irregularities in the size distribution and lateral shape. This, coupled with their relatively small average size, hinders their tendency to order into stacked clusters in solution. WAXS data for BCSC500 did not provide clear evidence of enhanced crystallinity upon annealing as PLLA crystals cannot thicken during this process due to the presence of side methyl groups. However, annealing may improve the lateral shape and size distribution of the crystals, contributing to the emergence of ordering in SAXS at 105 °C during heating. Even after a short seeding period at 104 °C, ordering is already discernible. At this stage, the crystal size should be relatively small and the number of crystals may not be extensive. Yet, the presence of ordering indicates that high temperatures favor its formation. Additionally, the disappearance of ordering upon cooling further supports the temperature-enhanced order formation in our system. Although one might argue that imperfections during cooling diminish ordering, the temperature still appears to play a significant role.

For BCSC1000, the solution is in the N phase, where the concentration of platelets is significantly higher than that in the isotropic phase. Upon heating, the system exhibits an annealing effect, sharpening the SAXS/WAXS peaks and revealing high-order peaks and intensity. This effect is likely due to the improved crystallinity and enhanced lateral interactions between the platelets in the concentrated N phase. Annealing also promotes the formation of larger, more ordered sheets. The presence of up to the ninth order of correlation strongly indicates the formation of lamellar domains within the N phase. During seeding and growth, memory effects may contribute to maintaining the ordering.

While the size difference of the two systems is the main reason for their initial phase behavior at room temperature (Figure 1a,b inset images of capillaries), this is not the primary reason for the disappearance of the order during cooling. Combining POM and SAXS results, it becomes clear that the ordering observed in BCSC500 via SAXS is due to the temperature-enhanced short-range order, which gradually dissipates upon cooling in the isotropic phase. In contrast, BCSC1000 in the nematic phase exhibits not only large-scale nematic order but also short-range correlation between plates, enabling the persistence of order after cooling.

## Conclusions

In summary, we have shown in this work that temperature is an important parameter in tuning the I–N transition in plate-like colloid systems. Polystyrene-*block*-poly(l-lactide) (PS-*b*-PLLA) truncated single crystals with mean sizes of 500 and 1000 nm were prepared by controlling the self-seeding temperatures. The I–N phase transition and structure evolution of BCSC suspensions were followed by real-time SAXS/WAXS and POM during heating, quenching, self-seeding, and final cooling. Both BCSC500 in the isotropic phase and BCSC1000 in the N phase showed enhanced ordering upon heating before the melting of the crystals. After seeding and crystal growth, both systems exhibited ordering. However, during the final cooling to room temperature, ordering disappeared for BCSC500 but persisted for BCSC1000.

Although both SAXS and POM revealed an increased order, discrepancies were observed. SAXS indicated intensified short-range correlations, whereas POM showed the formation of local nematic domains or tactoids. We propose three distinct ordering regimes to explain these observations: (a) At lower temperatures, large-scale nematic order emerges, visible in both SAXS and POM. However, lateral attraction between PLLA crystalline cores restricts the flexibility of BCSC plates, hindering further alignment. (b) At higher temperatures, increased thermal energy and hydrodynamic shear effects overcome these interactions, enabling greater platelet mobility and facilitating the formation of new, energetically favorable orientations. This enhanced short-range order with local nematic domains is detectable by both SAXS and POM. Nevertheless, due to increasing thermal energy, large-scale nematic order is disrupted in most regions of the solution. (c) At temperatures approaching melting, plates may form short-range clusters, which are exclusively observable by SAXS. In addition to the temperature-enhanced ordering, the annealing effect and memory effect in the polymer melt and crystals also contribute to the ordering.

## Supporting Information Available

Normalized SAXS profiles for BCSC1000 and normalized WAXS profiles for BCSC500 at different temperatures (PDF)
