# Supplementary material for: Temperature-Enhanced Ordering in Plate-like Semicrystalline Block Copolymer Single-Crystal Suspensions Studied by Real-Time SAXS/WAXS
Source: Langmuir. 2025 Feb 21;41(8):5009–20. doi: 10.1021/acs.langmuir.4c03853 (PMC11887440; doi:10.1021/acs.langmuir.4c03853)
Supplement: Supplementary file 1 — la4c03853_si_001.pdf [file la4c03853_si_001.pdf]

Supporting Information

Temperature Enhanced Ordering in Plate-like Semicrystalline Block Copolymer Single Crystal Suspensions Studied by Real-time SAXS/WAXS

Enyi Chi[a], *Haiying Huang*[a,b*], *Fajun Zhang*[c*], *Tianbai He*[a, b]

[a]State Key Laboratory of Polymer Physics and Chemistry, Changchun Institute of Applied Chemistry, Chinese Academy of Sciences, Changchun 130022, P. R. China

[b]University of Chinese Academy of Sciences, Beijing 100049, P. R. China

[c]Institut für Angewandte Physik, Universität Tübingen, Auf der Morgenstelle 10, 72076 Tübingen, Germany

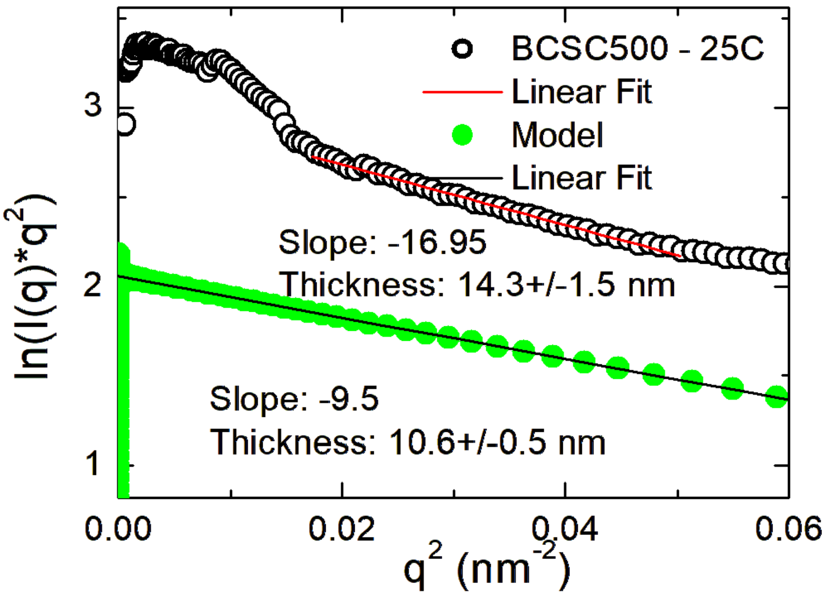

Figure S1: Modified Guinier plot for BCSC500 at the room temperature to determine the thickness of the plate-like crystal in solution. For disc-like particles in solution, the scattering intensity in the intermediate q range can be approximated as: $I(q) \sim q^{-2} \exp\left(-q^2 \frac{h^2}{12}\right)$, here h is the thickness of discs [1]. The thickness of the crystals is about 14.3 nm. In comparison, the Guinier analysis for the model form factor of a single layer disc with a thickness of 10 nm shown in Figure 2a gives a thickness of 10.6 nm.

Reference:

[1] Glatter, O., and O. Kratky. Small angle X-ray scattering. Academic Press: London, 1982.

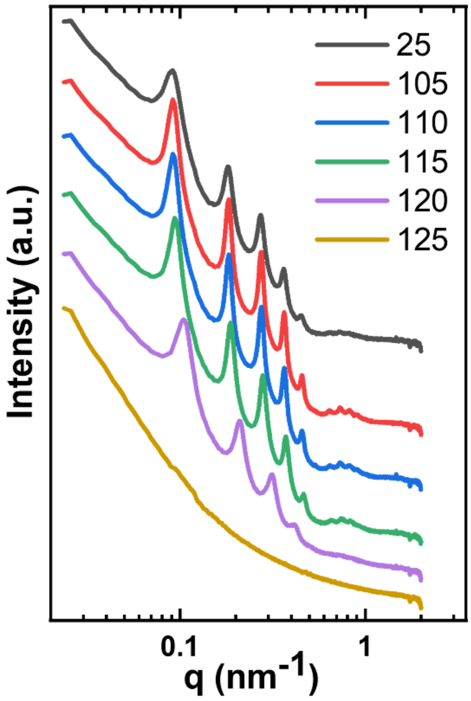

Figure S2: SAXS for BCSC1000 during heating from 25 to 130℃.

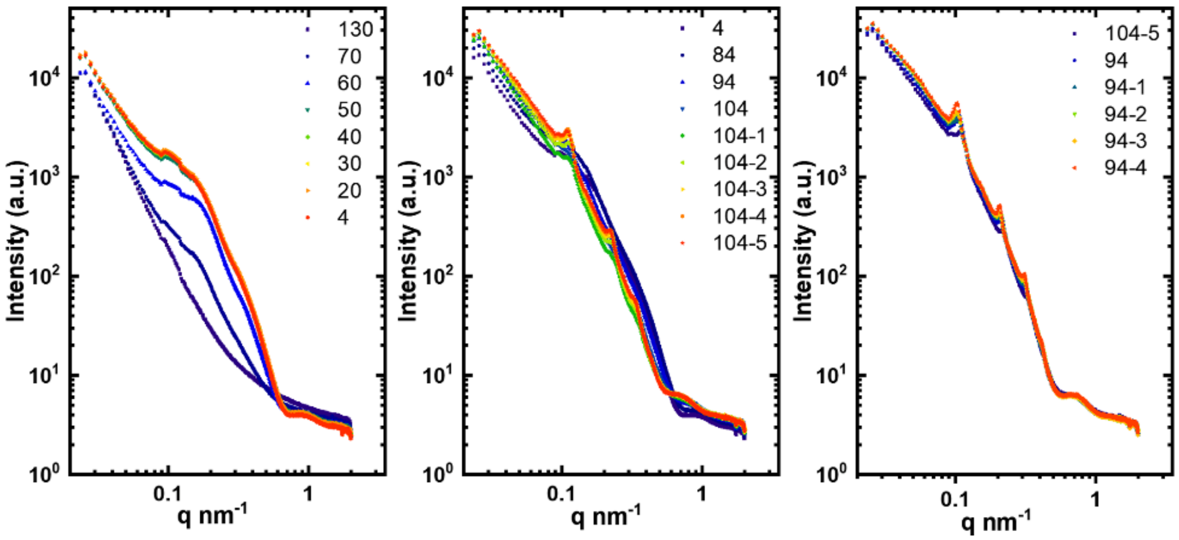

Figure S3. SAXS for BCSC500 during self-seeding and growth at 104 and 94℃, respectively.

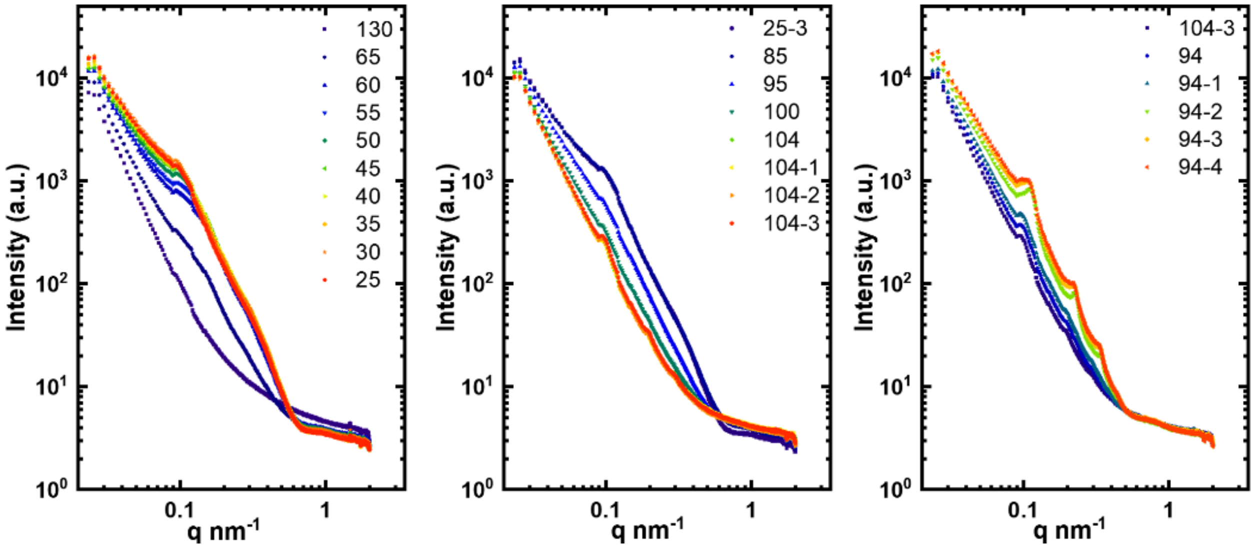

Figure S4. SAXS for BCSC1000 during self-seeding and growth at 104 and 94℃, respectively.

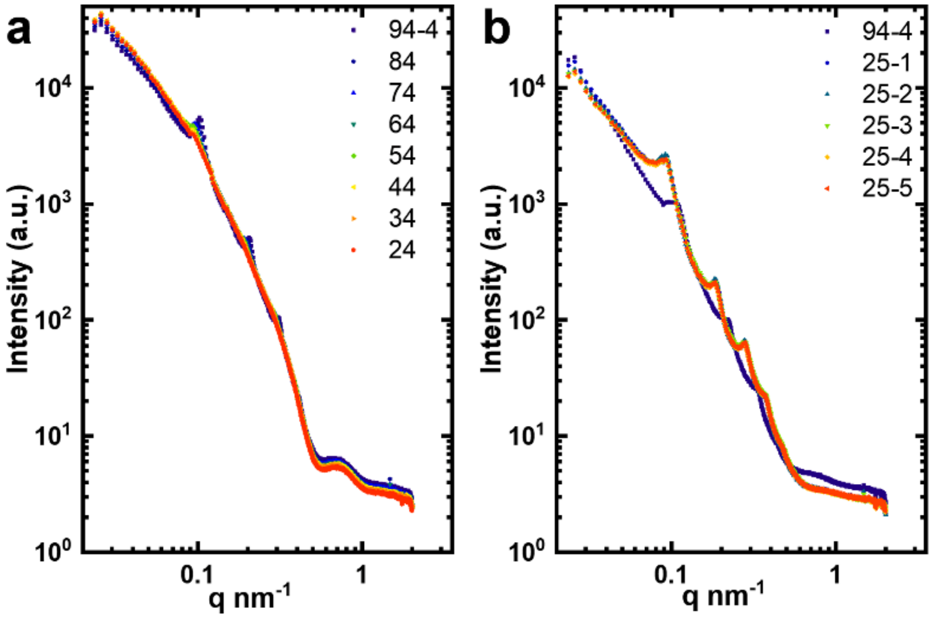

Figure S5. SAXS for BCSC500 (a) and BCSC1000 (b) during the final quench from 94 to 24 or 25 ℃.

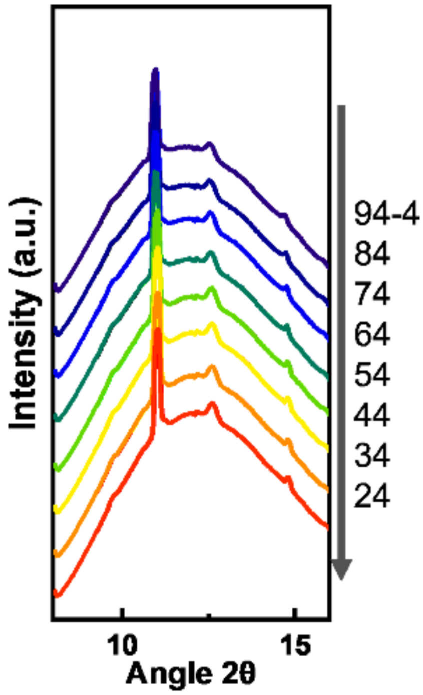

Figure S6. WAXS for BCSC500 during the final quench from 94 to 25℃.

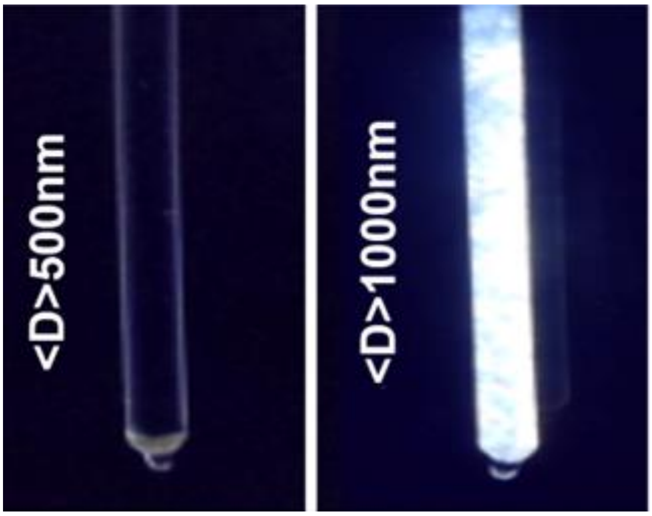

Figure S7. Photographs of sample capillaries under cross polarizer.
